# No selection on immunological markers in response to a highly virulent pathogen in an Arctic breeding bird

**DOI:** 10.1111/eva.12180

**Published:** 2014-06-26

**Authors:** Pierre Legagneux, Lisha L Berzins, Mark Forbes, Naomi Jane Harms, Holly L Hennin, Sophie Bourgeon, H G Gilchrist, Joël Bêty, Catherine Soos, Oliver P Love, Jeffrey T Foster, Sébastien Descamps, Gary Burness

**Affiliations:** 1 Département de biologie & Centre d’études nordiques, Université du Québec à RimouskiRimouski, QC, Canada; 2 Environmental and Life Sciences Graduate Program, Trent UniversityPeterborough, ON, Canada; 3 Department of Biology, Carleton UniversityOttawa, ON, Canada; 4 Department of Veterinary Pathology, University of SaskatchewanSaskatoon, SK, Canada; 5 Department of Biological Sciences, University of WindsorWindsor, ON, Canada; 6 Norwegian Polar Institute, Fram CentreTromsø, Norway; 7 National Wildlife Research Centre, Environment CanadaOttawa, ON, Canada; 8 Environment CanadaSaskatoon, SK, Canada; 9 Center for Microbial Genetics & Genomics, Northern Arizona UniversityFlagstaff, AZ, USA; 10 Department of Biology, Trent UniversityPeterborough, ON, Canada

**Keywords:** avian cholera, clutch size, common eider (*Somateria mollissima)*, immune traits, natural selection, *Pasteurella multocida*

## Abstract

In natural populations, epidemics provide opportunities to look for intense natural selection on genes coding for life history and immune or other physiological traits. If the populations being considered are of management or conservation concern, then identifying the traits under selection (or ‘markers’) might provide insights into possible intervention strategies during epidemics. We assessed potential for selection on multiple immune and life history traits of Arctic breeding common eiders (*Somateria mollissima*) during annual avian cholera outbreaks (summers of 2006, 2007 & 2008). We measured prelaying body condition, immune traits, and subsequent reproductive investment (i.e., clutch size) and survival of female common eiders and whether they were infected with *Pasteurella multocida,* the causative agent of avian cholera. We found no clear and consistent evidence of directional selection on immune traits; however, infected birds had higher levels of haptoglobin than uninfected birds. Also, females that laid larger clutches had slightly lower immune responses during the prelaying period reflecting possible downregulation of the immune system to support higher costs of reproduction. This supports a recent study indicating that birds investing in larger clutches were more likely to die from avian cholera and points to a possible management option to maximize female survival during outbreaks.

## Introduction

In natural populations, unpredictable environmental perturbations can occur from changing climatic conditions, habitat degradation, and introduction of predators or epidemics. Such occurrences can offer useful, quasi-experimental opportunities to explore and potentially uncover intense natural selection on morphological (Brown and Brown 1998; Grant and Grant 2002), physiological (Wilcoxen et al. 2010), or other fitness-related traits (Carroll et al. 2007). In the case of disease, investment into relevant traits of immunity and subsequent survival are predicted to relate to one another (Norris and Evans 2000; Møller and Saino 2004; Graham et al. 2010). Natural selection mediated by disease has received increasing attention, and studies to date, have suggested directional selection on particular immune traits (Altizer et al. 2003; Wilcoxen et al. 2010; Woodhams et al. 2011).

Selection on life history traits is also expected. The trade-off between current reproduction and survival is one of the most prominent topics in ecology and life history theory (Williams 1966; Stearns 1992; Ricklefs and Wikelski 2002). Timing of reproduction and breeding effort are expected to be direct drivers of an individual’s fitness, but can also affect subsequent survival and/or future reproduction (Faaborg et al. 2010). Measuring these types of trade-offs is challenging, and they are often only revealed through experimental manipulation (Gustafsson et al. 1996; Sheldon and Verhulst 1996). However, to our knowledge, no studies have yet investigated trade-offs between immune traits, reproductive investment and survival during a disease outbreak, when they are most likely to be detected.

When the population being considered is of management or conservation concern, it is important to understand how phenotype translates into increased exposure and infection and into reduced survival. Such an understanding might help evaluate potential management options. For example, at the outset of an epidemic, the finding that some individuals carry genetic or physiological markers of resistance might be followed by assessing the proportion of such genotypes/phenotypes in the population as an index to determine whether the population is naïve or experienced with the disease agent. Management options might vary from no intervention to reducing harvest depending on the degree to which the populations are naïve.

Avian cholera, caused by the bacterium *Pasteurella multocida*, constitutes a major threat for free-living birds, particularly in colonially nesting species (Friend et al. 2001), because of the magnitude of losses it can cause (Friend et al. 2001; Descamps et al. 2011a, 2012). Common eiders (*Somateria mollissima*), a long-lived waterfowl species nesting at a low Arctic colony at East Bay Island, Nunavut, Canada, have experienced annual avian cholera outbreaks during the breeding period since 2005 (Descamps et al. 2012). In some years, survival of both adult females and ducklings has been dramatically reduced (>30% and >90%, respectively: Descamps et al. 2009, 2011a). Moreover, females that laid larger clutches were more likely to die during outbreaks than females with smaller clutches (Descamps et al. 2009). Importantly, clutch size and lay date are related to prelaying body condition in this species (i.e., endogenous reserves: Descamps et al. 2011b), which is in turn closely associated with immunity and stress in female eiders which fast during incubation (Bourgeon et al. 2006). Thus, immune function may provide a causal link between an individual’s state and subsequent survival (Hanssen et al. 2003; Bourgeon et al. 2006).

Here, we take advantage of a series of annual outbreaks of avian cholera occurring in a closely monitored nesting colony of common eiders to explore and potentially uncover adaptive variation among females in immune and immune-related traits. We first tested whether six measures of immunity varied between prelaying female eiders that were asymptomatically infected with *P. multocida* (and thus, possible carriers) versus uninfected females. Secondly, we tested for either directional or stabilizing selection on survival during outbreaks in relation to prelaying immune traits. Finally, because females that invest more in reproduction appear more likely to die from avian cholera during outbreaks (Descamps et al. 2009), we tested whether females with larger clutches had lower investment in immunity. We also compared immune traits between prelaying and incubating females. According to life history theory (Norris and Evans 2000), we expected to find a negative relationship between immune traits in incubating females and clutch size (i.e., a trade-off between reproductive investment and physiological state).

## Material and methods

### Study site and species

This study was conducted on East Bay Island (64°02′N, 81°47′ W) in the East Bay Migratory Bird Sanctuary, Nunavut, Canada from 2006 to 2008, during major outbreaks of avian cholera (Descamps et al. 2009). No previous selection due to avian cholera was expected because the nesting island has been monitored intensively since 1996 and avian cholera was first confirmed in 2005, with 2006 being the first year with high mortality caused by this disease. Adult females were captured, blood sampled, measured, and banded during the prelaying period as part of a long-term mark–recapture program. All female common eiders captured were marked with a unique color and shape combination of two temporary plastic nasal markers (Juno Inc., Minneapolis, MN) so that nasal-tagged individuals could be identified on nests. In 2007 and 2008, oral and cloacal swabs were also collected from prelaying adult female eiders at capture to determine whether birds were asymptomatically infected with *P. multocida*. Information on clutch size for nasal-tagged birds was available for a relatively low sample size (*n* = 46).

To assess immune traits during the incubation period, 23 and 36 incubating hens were captured in 2007 and 2008, respectively, using nest traps. Hens were bled immediately following capture. These females were randomly chosen from those not marked to limit interference in the long-term demographic monitoring. We are confident that females captured before and during incubation are representative of the entire population and generate two comparable, unbiased groups. Laying dates for the two groups were not statistically different (*t* test = 1.75, df = 74.9, *P* = 0.08).

Following late season surveys of the entire island in all 3 years, birds were classified as dead if their carcass was found on site during or after incubation, or classified as alive if they were resighted in up to 3 years following sample collection. Females are highly philopatric, and intensive resighting efforts were carried out each spring to ensure high resighting probabilities (Descamps et al. 2011b). A total of 7.5% of females that were neither resighted nor found dead were discarded from analyses, allowing us to focus analyses on avian cholera-induced mortality. Including those females (as dead individuals) had no effect on the results (analyses not shown). Carcasses were submitted to the Canadian Cooperative Health Centre for necropsy, and avian cholera was diagnosed as the cause of death each year in all cases, based on gross and histopathological findings and bacteriology (CCWHC database). This study adhered to guidelines of the Canadian Council on Animal Care, and all protocols were reviewed and approved by University Animal Care Committees Saskatchewan: 20100063; Windsor: AUPP 11-06; Trent: 07032, and by Environment Canada’s Animal Care Committee (Protocol Numbers: EC-PN-07-008 (2007), EC-PN-08-026 to EC-PN-11-026 (2008 to 2011).

### Prelaying *Pasteurella multocida* infection status

We used a recently developed real-time PCR technique (Corney et al. 2007) to detect the presence of *P. multocida* DNA from two swab samples (oral and cloacal) collected from female eiders during capture in 2007 and 2008. We considered an individual to be infected with *P. multocida* if at least one of the samples from that individual was positive (further details are provided in the Appendix S2). All swabs were collected prior to or within 2 days following the detection of the index case of avian cholera on the island each year, and all birds sampled were apparently healthy. It is possible that birds with PCR-positive samples were carriers of *P. multocida* (asymptomatically infected birds that can serve as potential reservoirs for initiating outbreaks: Samuel et al. 2005); however, we were not able to differentiate between true carrier birds and acutely infected birds not yet showing clinical signs. Furthermore, the PCR results suggest that a bird is infected with *P. multocida*, but do not provide any further information regarding the strain, serotype, or pathogenicity of the organism, or whether or not it is the same strain causing subsequent outbreaks. Thus, we refer to PCR-positive birds as asymptomatically infected birds rather than as carrier birds.

### Immune analyses

We maximized our ability to adequately cover immune system complexity (Matson et al. 2006) by simultaneously considering and quantifying multiple immune indices including heterophile:lymphocyte ratio (H:L ratio), basophils, avian immunoglobulin (IgY), complement (Comp), natural antibody (NAb), and haptoglobin (Hp; assay details provided in the Appendix S3). Infection from *P. multocida* impairs inflammation (Christensen and Bisgaard 1997) and potentially hinders the activation of lymphocytes (Gong et al. 2013). Moreover, *P. multocida* bacteria express a capsule on their surface, which plays an important role in pathogenesis, prevents phagocytosis (Christensen and Bisgaard 1997), and is protective against complement activity (Chung et al. 2001). The immune traits we chose to measure thus gave us information on both innate and acquired immune systems. Plasma IgY and Hp were obtained from blood samples taken within 3 min of capture and handling. For the remaining immune traits, blood sampling occurred within 10–140 min of capture. For incubating females, the following immune traits were measured: Hp, IgY, NAb, and Comp.

### Statistical analyses

To examine differences in immune responses between asymptomatically infected and uninfected birds, we used a permutation test. For each parameter, a null distribution of the test statistic was approximated by a Monte Carlo resampling (10 000 Monte Carlo replications) to control for unbalanced sample size. We then estimated the strength and mode of selection (linear and nonlinear selection) using multiple regressions (Blows and Brooks 2003). We regressed relative fitness (here mortality rate using individuals recovered dead on site in a given year, coded as 1, versus individuals resighted alive for up to 3 years, coded as 0) on standardized log-transformed immune parameters. We first calculated the selection differential (S, the net effect of selection on a trait: difference between the mean value of the trait in all birds sampled and the value of the trait in only the survivors). We used *t* tests to assess statistical significance of S. For each immune trait, we then used mixed model analyses with a binomial distribution of errors (with a logit link function) that included linear and quadratic terms (to account for possible directional or stabilizing selection, respectively) with year as a random effect to account for the structure of the data that included recaptures. This approach is similar to Stjernman et al. (2008). Prelaying body condition, as measured by body mass (the best proxy of condition in our case, see Descamps et al. 2011b), was used to control for state at capture. Since body size and immune response can be related (Christe et al. 2000), we also used body size (see details of calculations in Appendix S1) as an explanatory covariate in the analyses. The ratio between residual deviance and residual degrees of freedom was calculated for all models and was close to one (range: 0.963–1.011) revealing no over-dispersion (Crawley 2012). Because all parameters were not necessarily measured for a given individual, considering multiple immune parameters in a single model would have reduced sample size to only 57 individuals. To maximize sample size, results for each immune parameter were examined and are presented separately in the main document. In the appendix, we nonetheless present selection analyses (using *n* = 57 individuals) from a principal component analysis to reduce the number of immune variables to two orthogonal components using varimax rotation (*R* package psych) following Buehler et al. (2012).

For each immune trait, we used a backward stepwise model selection procedure to get a minimal adequate model (Crawley 2012), starting with the global model and subsequently removing all nonsignificant terms (*P*-values > 0.1, allowing us to detect marginal effects). Only two-way, biologically relevant interactions were considered (between immune parameter and body size or condition). Because some immune traits can severely be impacted by handling stress (Chin et al. 2013), handling time was entered as a covariate in the models. For H:L ratio, basophils, NAb and Comp, handling time was retained as a covariate. Because clutch size was available for a relatively low number of monitored individuals (both for females captured during the prelaying period and for females captured during incubation), we performed additional analyses to test for relationships between immune traits and clutch size using linear mixed models with year as a random effect. We compared the immune traits of prebreeding (with known laying dates) and incubating females measured in 2007 and 2008 using permutation tests (see above).

## Results

Of the 351 individuals tested for the presence of *P. multocida* DNA using PCR techniques, 26 tested positive for infection with avian cholera. Infected birds had a greater level of Hp compared to uninfected birds (permutation test: χ^2^ = 18.9; *P* < 0.001, *n* = 190, with mean = 0.31 ± 0.03 SE and 0.69 ± 0.10 SE in noninfected and infected birds, respectively). However, infection status was not associated with any other indices of immunity (all χ^2^ < 3.03 and all *P* > 0.22, except for IgY, which showed a marginal effect: χ^2^ = 5.61; *P* = 0.06 with PCR-positive birds presenting slightly lower IgY values than PCR-negative birds: mean = 0.61 ± 0.05 SE vs 0.72 ± 0.03). Furthermore, infection status (measured during the prelaying stage) did not predict the likelihood of female mortality by the end of the breeding season (mean mortality rate was 0.77 ± 0.05 and 0.86 ± 0.02, respectively; GLMM: β = ± 0.78; *P* = 0.50).

**Figure 1:**
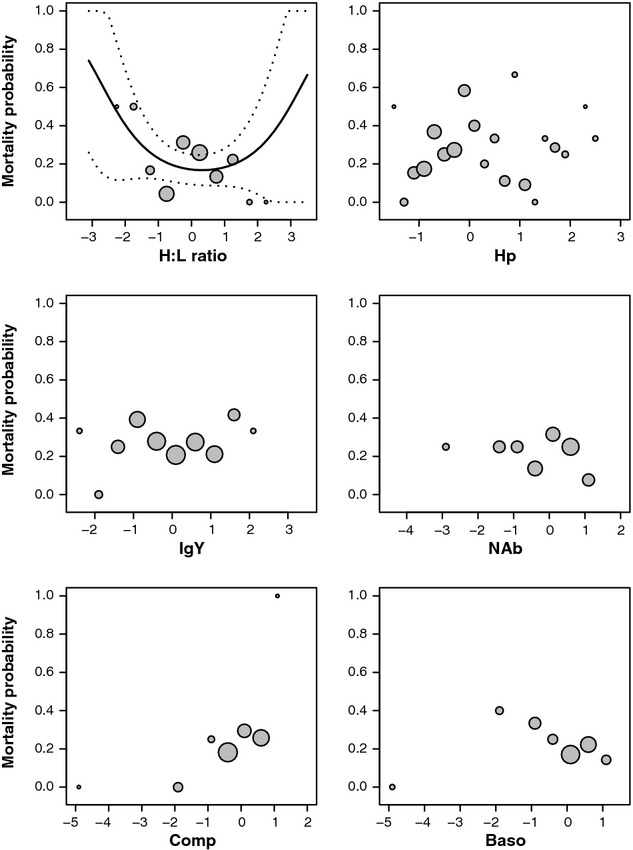
Probability of mortality in relation to various standardized log-transformed immune traits measured in female common eiders prior nesting. The fitted logistic model (black line) as well as its confidence interval at 95% (dotted line) is shown only when retained in Table 1. Gray circle sizes are proportional to log (*n*) and represent raw data.

We found either no evidence or weak support for natural selection on measured immune traits (Table 1). Similar results were obtained using an information theoretical approach (Table S1). Even when a given immune trait predicted the probability of mortality (i.e., H:L ratio), relationships were associated with large standard errors or depended on outliers (Fig. 1 and Figure S1). Similarly, results from PCA gave poor support to selection (β = −0.08 ± 0.38; *P* = 0.83, and β = −0.55 ± 0.37; *P* = 0.14, *n* = 57 with the first and second axis of the PCA, respectively, Figure S1).

**Table 1:** Results from Generalized Linear Mixed Models with a binomial distribution of errors exploring association between mortality probability and different immune parameters. Similar models were performed for each immune trait. Immune trait^2^ represented the quadratic term. Body size and condition (see ESM for details) were also entered in the initial model. *S* is the selection differential. Slope (selection coefficient: *β*), standard errors (SE) and statistic (*Z* and *P* values) are given. Sample size (*N*) and Nagelkerke Pseudo *R*^2^ are given under brackets for each immune covariate. Only results from the best model are presented.

	HL ratio (*N* = 103; *R*^2^ = 0.03; *S* = −0.02; *P* = 0.80)				Hp (*N* = 181; *R*^2^ = 0; *S* = 0.01; *P* = 0.81)				IgY (*N* = 238; *R*^2^ = 0; *S* = 0.01; *P* = 0.83)			
	*β*	SE	*Z*	*P*	*β*	SE	*Z*	*P*	*β*	SE	*Z*	*P*
Intercept	−2.07	0.60	−3.45	<0.001	−1.02	0.24	−4.32	<0.001	−1.01	0.19	−5.42	<0.001
Immune trait	−0.20	0.24	−0.85	0.40	–	–	–	–	–	–	–	–
Immune trait^2^	0.23	0.12	1.83	0.07	–	–	–	–	–	–	–	–
Size	–	–	–	–	–	–	–	–	–	–	–	–

	NAb (*N* = 114; *R*^2^ = 0.06; *S* = −0.01; *P* = 0.87)				Complement (*N* = 114; *R*^2^ = 0.07; *S* = −0.05; *P* = 0.18)				Basophils* (*N* = 101, *R*^2^ = 0; *S* = 0.04; *P* = 0.27)			
	*β*	SE	*Z*	*P*	*β*	SE	*Z*	*P*	*β*	SE	*Z*	*P*
Intercept	−1.25	0.67	−1.88	0.06	−1.25	0.67	−1.88	0.06	−1.57	0.52	−3.02	0.002
Immune trait	–	–	–	–	–	–	–	–	–	–	–	–
Immune trait^2^	–	–	–	–	–	–	–	–	–	–	–	–
Size	−0.29	0.15	−2.01	0.04	−0.29	0.15	−2.01	0.04	–	–	–	–

* Null model (intercept only) was elicited for Eosinophils and monocytes with similar sample size.

The effect of body size on mortality (found in models testing for NAb and Comp) was also weak and did not remain when the complete dataset was analyzed (i.e., including all sampled birds whatever the immune traits measured: β = −0.06 ± 0.09; *P* = 0.55, *n* = 317). Body condition was not retained to explain mortality for any of the tested parameters.

**Figure 2:**
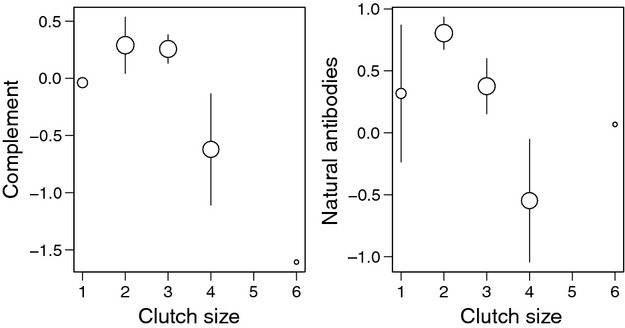
Relationship between complement or natural antibodies (standardized log-transformed) and clutch size in prelaying female common eiders. Both negative relationships are significant (see results). Bars represent SE; dot sizes are proportional to log (*n*).

**Figure 3:**
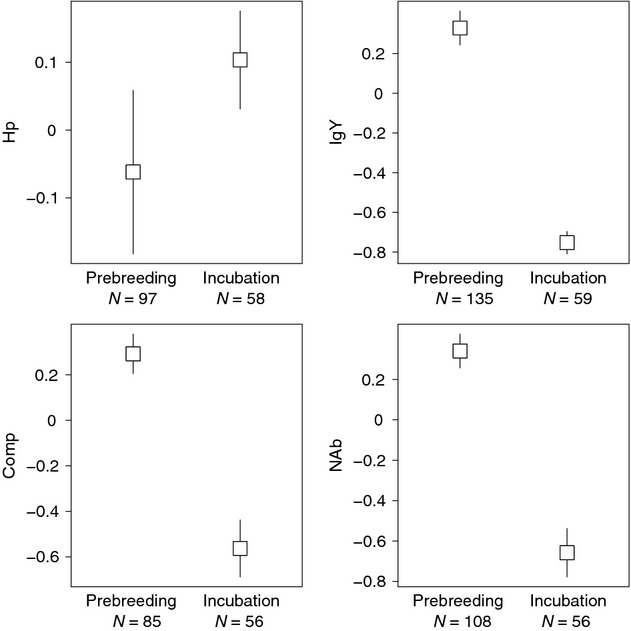
Mean (+SE) values of haptoglobin, IgY, complement, and natural antibody (standardized log-transformed) in prelaying and incubating female common eiders captured on East bay Island (Nunavut) in 2007 and 2008.

Females incubating larger clutches had lower NAb and Comp levels during the prelaying period compared with females incubating smaller clutches (*F*_1,43_ = 4.35; *P* = 0.04 and *F*_1,43_ = 3.97; *P* = 0.05, respectively, Fig. 2). However, clutch size was not related to any of the other prelaying immune traits (All *F* < 1.74, all *P* > 0.19; *n* ≥ 46). We found that incubating females had lower NAb, Comp and IgY and higher Hp than prelaying females (permutation tests: all χ^2^ > 39.49 and all *P* < 0.001; Fig. 3). Finally, in females captured during incubation, there was no relationship between clutch size and immune traits (All *F* < 1.15, all *P* > 0.29; *n* > 55, Figure S2).

## Discussion

Our results revealed no stabilizing or directional selection on any of the immune traits measured in the presence of a highly virulent disease in a population of Arctic breeding birds. These results point to the possibility of the population being naïve to Avian cholera, a practical consideration which is also suspected based on long-term monitoring of common eiders at that site. Our results are in contrast to the very few studies that have investigated selection on immune traits during epidemics (Wilcoxen et al. 2010; Woodhams et al. 2011). In birds, only one study reported directional selection on an immune trait (bacterial killing ability) during a suspected outbreak of eastern equine encephalitis (Wilcoxen et al. 2010). Florida scrub jays (*Aphelocoma coerulescens*) that survived the epidemic had greater bacterial killing ability and were in better body condition than individuals that died (Wilcoxen et al. 2010). In the present study, although infected birds responded to infection (see below), prebreeding immune investment had no major repercussions on subsequent probability of mortality due to avian cholera. The difference between our results and those of previous studies might reflect the degree to which the populations under study had previous experience with the same or similar disease agent.

We found that asymptomatically infected birds (i.e., apparently healthy eiders with *P. multocida* DNA in their oral cavity or cloaca) exhibited elevated Hp levels, an acute phase protein that scavenges hemoglobin in the event of intravascular or extravascular hemolysis (Quaye 2008). Concentrations of Hp increase in response to recent infection (Quaye 2008) and often represent the onset of a nonspecific immune response (Matson et al. 2012). Although it could be argued that an increase in Hp could represent a maladaptive over-investment in immunity, we argue this is not the case because this immune trait was the only one to change with respect to infection status. It thus suggests birds testing positive for *P. multocida* had been recently exposed and may have been dealing with acute infections. However, prelaying infection with *P. multocida* did not predispose nor protect birds from dying of avian cholera once outbreaks began.

PCR-positive test results in apparently healthy birds do not distinguish between true carrier birds and acutely infected birds. Because there is the potential for these scenarios to result in two very different immunological states, we might expect differences in their immune traits and fate. Thus, if both types of individuals are represented in our PCR-positive results, it is possible that any relationships between true carrier status and immune function (or survival) or between acute infection and immune function (or survival) may have been masked. No selection on immune traits also means that there were no real immunological markers for disease susceptibility or mortality. Thus, we could not assess the expected magnitude of the outbreak at the start of the epidemic even if we had obtained our immunological results early on. One concern in such studies is masking of patterns.

We propose three explanations for the apparent lack of selection on immune traits observed here. First, immune indices measured during the prelaying period may not reflect relevant immunocompetence approximately 1 month later (mainly during incubation) when most individuals died from virulent *P. multocida*. The observed decrease in IgY over the course of incubation has previously been reported in common eiders (Bourgeon et al. 2006) as well as in other species (Hõrak et al. 1998) in the absence of disease outbreaks. Here, we reported a major shift in a variety of immune traits during incubation, including IgY, suggesting that significant immunological changes occur during this key reproductive stage. Although we are confident that individuals captured during the prelaying period are representative of the monitored population, repeated sampling would have been required to examine whether the magnitude of change from prelaying to incubation in immunological traits was both variable and predictive of survival.

Indeed, immune responses measured during one life history stage can carryover and influence survival during subsequent life history stages (Råberg et al. 2009; Wilcoxen et al. 2010). For example, an injection of nonpathogenic antigens in incubating common eiders resulted in a significant reduction in return rates and survival (measured 1 year after injection; Hanssen et al. 2004). Another problem inherent with our study model is that we could not control for age. Knowing the number (and effort) of past reproductive events would have enabled us to investigate long-term carryover effects of the costs of reproduction (Nordling et al. 1998).

A second interpretation is that the bacteria are so virulent that most females died upon exposure, regardless of their immunity. In domesticated species, similar massive die-offs have occurred without any evidence that immune measures differed between survivors and nonsurvivors (Christensen and Bisgaard 2000; Li et al. 2000). In a similar vein, Descamps et al. (2011a) reported that outbreaks of avian cholera caused by highly virulent strains of *P. multocida* removed the expected stabilizing effects of hatching date and directional effects of hatching mass on recruitment. One consideration is that a conservative immunological readiness combined with genetic variability at recognition loci would likely be optimal (Viney et al. 2005), but might result in such massive die-offs to some pathogens if few individuals have appropriate loci and many individuals have conservative immunity.

Finally, it could be that the immune traits investigated, despite their widespread use in eco-immunology, were not those most important to determining survival in the face of an avian cholera epidemic. Given the complexity of the avian immune system with multiple regulation pathways (Schat et al. 2014), information on immune responses is often incomplete and limited to a relatively limited number of techniques (Adelman et al. 2014). This is particularly the case in remote field locations such as ours, which constrains usability of techniques.

In our study colony, and during the same period as this study, Descamps et al. (2009) showed that females with larger clutches were more likely to die from avian cholera. Fitness costs of greater reproductive investment could result in immune modification (Martin et al. 2001), longer exposure time or closer proximity to avian cholera outbreaks. We previously argued that timing of infection (and probably exposure time to the disease) was unlikely to explain mortality from avian cholera. Alternatively, in the current study, we found relative support for a cost of reproduction as females with larger clutches had an overall lower level of natural antibodies and complement. This finding has twofold relevance. First, our measures of immunity are relevant in examining trade-offs (see Immune analysesImmune analyses section in Methods), even if apparently irrelevant as markers of mortality. Second, this relationship was significant for two key components of innate immunity that can, respectively, neutralize infection and lead to the rupture of pathogens once activated (Ochsenbein and Zinkernagel 2000). Similar results were found in at least two bird species using brood or clutch size manipulation; individuals with increased parental care exhibited lower levels of antibodies or complement (Deerenberg et al. 1997; Berzins et al. 2011).

Because immune traits considered here do not seem to mediate any prominent mechanisms affecting survival, we conclude that eiders may be more affected by avian cholera because of egg production and/or incubating larger clutches (Descamps et al. 2009) than by a slight reduction of their specific immunocompetence at the onset of reproduction. This reflects possible downregulation of the immune system to support higher costs of reproduction (Norris and Evans 2000). As we have confirmed that a significant reduction in immune responses occurs during incubation, the individual effects of even slightly lower levels of innate immunity could be magnified at future life history stages with serious potential repercussions on survival during a novel disease outbreak. This study highlights possible physiological trade-offs between self-maintenance and reproduction, further suggesting that links between immunocompetence and fitness are highly complex, making them difficult to document and disentangle in free-living systems (Linden and Møller 1989; Gustafsson et al. 1996; Love et al. 2008).

## Practical applications

As mortality appears mediated by investment in reproduction and outbreaks typically occur on the breeding grounds, managers may consider encouraging subsets of females to abandon nesting attempts. This would have the dual effect of reducing the potential for density-dependent transmission if nonnesting females leave colonies as well as potentially allowing some females to avoid exposure or recover from infection if exposed without paying further costs of reproduction. Such considerations merit further investigation.

## Data archiving

Data for this study are available in the Dryad Digital Repository: http://dx.doi.org/10.5061/dryad.7d0k4.

## Supporting Information

**Table S1.** Model selection based on information-theoretic approach to assess the effect of immune parameters on mortality. **Figure S1.** Probability of mortality in relation to the first two components of a Principal Component Analysis. **Figure S2.** Relationship between immune parameters and clutch size in incubating common eider females. **Appendix S1.** Body condition and body size estimates. **Appendix S2.** Methods of real-time PCR to detect *Pasteurella multocida* in eider swab samples. **Appendix S3.** Detailed methods used to measure immune indices.
