# Supplementary material for: No selection on immunological markers in response to a highly virulent pathogen in an Arctic breeding bird
Source: Evol Appl. 2014 Jun 26;7(7):765–73. doi: 10.1111/eva.12180 (PMC4227857; doi:10.1111/eva.12180)
Supplement: Supplementary file 1 — Table S1. Model selection based on information-theoretic approach to assess the effect of immune parameters on mortality. Figure S1. Probability of mortality in relation to the first two components of a Principal Component Analysis. Figure S2. Relationship between immune parameters and clutch size in incubating common eider females. Appendix S1. Body condition and body size estimates. Appendix S2. Methods of real-time PCR to detect Pasteurella multocida in eider swab samples. Appendix S3. Detailed methods used to measure immune indices. [file eva0007-0765-sd1.docx]

**Table S1.** ΔAICc of 20 competing models performed for each of the 6 immune traits explaining mortality rates (binomial distribution). Similar models were ran for each covariate (Cov). Cov^2^ represented quadratic term. Body size and condition were additional covariates. The minimum AICc value and the pseudo R^2^ for the most parsimonious model for each immune trait are also presented. Except for Hp and IgY, handling time was considered in all models. For Hp and IgY, number of parameters is thus K-1.

|  | **K** | **HL ratio** | **Hp** | **IgY** | **NAB** | **Comp** | **Baso** |
| --- | --- | --- | --- | --- | --- | --- | --- |
| Null | 3 | 0 | 0 | 0.84 | 1.83 | 1.83 | 0 |
| Cov2 | 5 | 0.5 | 1.72 | 4.75 | 5.98 | 5.29 | 0.58 |
| Size | 4 | 0.81 | 0.69 | 1.75 | 0 | 0 | 0.81 |
| Cov | 4 | 1.6 | 1.43 | 2.84 | 3.96 | 3.48 | 1.99 |
| Size+Cov2 | 6 | 1.6 | 2.95 | 5.61 | 3.82 | 3.41 | 1.31 |
| Cond*Cov2 | 7 | 1.73 | 4.67 | 6.22 | 9.63 | 8.94 | 4.96 |
| Cond | 4 | 2.02 | 1.1 | 0.19 | 3.34 | 3.34 | 2.02 |
| Cond+COv2 | 6 | 2.2 | 2.67 | 4.12 | 7.58 | 6.96 | 2.8 |
| Size+Cond | 5 | 2.49 | 1.19 | 0 | 1.08 | 1.08 | 2.49 |
| Size+Cov | 5 | 2.5 | 2.3 | 3.8 | 2.07 | 1.51 | 2.9 |
| Cond+Size+Cov2 | 7 | 2.78 | 3.35 | 3.85 | 5.13 | 4.69 | 3.37 |
| Size+Cond*Cov2 | 8 | 2.81 | 5.32 | 5.89 | 7.19 | 6.74 | 5.55 |
| Cond+Cov | 5 | 3.56 | 2.49 | 2.12 | 5.49 | 5.1 | 4.03 |
| Cond*Cov | 6 | 3.84 | 4.43 | 4.21 | 7.5 | 7.06 | 6.03 |
| Size*Cov2 | 7 | 3.9 | 5.09 | 7.12 | 3.82 | 4.07 | 3.59 |
| Size+Cond+Cov | 6 | 4.09 | 2.81 | 2.01 | 3.22 | 2.69 | 4.6 |
| Size*Cov | 6 | 4.71 | 4.42 | 5.52 | 2.98 | 1.84 | 4.84 |
| Size+Cond*Cov | 7 | 4.72 | 4.71 | 4.03 | 5.26 | 4.72 | 6.6 |
| Cond+Size*Cov2 | 8 | 5.11 | 5.51 | 5.68 | 5.2 | 5.19 | 5.7 |
| Size*Cov+Cond | 7 | 6.33 | 4.94 | 3.97 | 4.1 | 2.89 | 6.63 |
|  |  |  |  |  |  |  |  |
| minimum AICc value |  | 109.63 | 212.91 | 277.89 | 119.37 | 119.37 | 109.63 |

**Figure S1.** Probability of mortality in relation to the first two components of a Principal Component Analysis (RC1 and RC2). The fitted logistic models (black line) as well as its confidence interval at 95% (dotted line) are shown. Grey circle sizes are proportional to log (N) and represents raw data. Biplot of the PCA is presented at the bottom. Values of RC1 and RC2 scores for each individual are represented with black dots. Gray arrows represent immune traits scores. The six variables had loadings > 0.49 on one of the first two axis (RC1 or RC2). RC1 and RC2 explained 27.4% and 22.1 % of total variation in the data respectively. SS loadings were 1.52 and 1.44 for RC1 and RC2 respectively.

**Figure S2.** Relationship between complement (Comp), Natural Antibodies (NAb), Immunoglobulin Y (IgY), haptoglobin (Hp) and clutch size in incubating common eider females. Bars represents SE, dot sizes are proportional to log (N). All immune traits were standardized log-transformed.

**Appendix S1. Body condition and body size estimates**

We restricted the analyses to birds captured during the pre-laying period in order to compare body condition and avoid any effect of egg laying on body mass ([Sénéchal et al. 2011](#_ENREF_19)). To do so, we excluded individuals captured after the date at which >2.5 % of the females started laying in the colony. Individuals with known laying dates (obtained from colony monitoring) were subsequently added if laying date was < to capture date (with a buffer of 3 days to account for potential error on laying date estimation). This procedure was done for each year separately. Because body mass alone is a better predictor of condition than mass corrected for body size in this species ([Descamps et al. 2011](#_ENREF_7)), we used OLS residuals of mass against capture date as an index of condition to control for possible mass increase during the pre-laying period. Body size was assessed using a principal component analysis on morphometric measurements (culmen, bill, tarsus and head lengths). The four variables had loadings ranging from 0.34 to 0.56 on the first axis (PC1), which explained 58.6% of total variation in the data. We used individual PC1 scores as a measure of body size.

**Appendix S2. Detailed methods used to detect carrier birds**

Oral and cloacal swab samples were collected from apparently healthy female common eiders (N=351) in 2007 and 2008, prior to detection of the index case of avian cholera on the island each year. Samples were collected using sterile polyester-tipped applicators (Puritan Medical Products Co., Guilford, ME) and stored individually in 2 mL polypropylene cryogenic vials (Thermo Scientific Inc., Rochester, NY) containing 1 mL of sterile Trypticase Soy Broth with 15% (v/v) glycerol (TSB glycerol) All swabs were immediately frozen at -196 °C in a dry-shipper liquid nitrogen tank (model SC 20/12V, MVE Vapour Shipper, MVE Bio-Medical Division, Chart Industries, Inc., Burnsville, MN) for transport to the Center for Microbial Genetics and Genomics (MGGen, Flagstaff, Arizona, USA). Samples were stored at -80 °C until analysis.

A previously reported 5’ Taq nuclease PCR assay ([Corney et al. 2007](#_ENREF_6)) was used to detect a sequence of the 16S rRNA gene of *P. multocida* in eider oral and cloacal swab samples with the following modifications. Cloacal and oral swab samples in TSB glycerol were thawed at room temperature for 1 hour. DNA was extracted from each sample using the following Chelex extraction protocol. Each swab sample was vortexed for 15-30 seconds. 5 uL of TSB glycerol from each sample was added to 60 uL of 5% Chelex solution (Chelex 100, Biorad, Hercules, California, USA), gently mixed, incubated at 95°C for 20 min, allowed to return to room temperature, and centrifuged at 4000 rpm for 1 min. *P. multocida* DNA was amplified in 10uL PCR mixtures containing 4 uL of template DNA, 4.89 uL mastermix (TaqMan Universal Mastermix, Invitrogen, Life Technologies, Grand Island, New York, USA), 0.5 uL forward and reverse primer at 10 uM (Sigma-Aldrich, Oakville, Ontario, Canada), 0.1 uL of TaqMan probe at 10 uM (Applied Biosystems, Life Technologies Inc, Grand Island, New York, USA), and 0.01 uL Taq (Platinum Taq, Invitrogen, Grand Island, New York, USA). An ABI7900 Real-Time PCR machine (Applied Biosystems, Grand Island, New York, USA) was used for all reactions with following cycling protocol: 40 cycles of 50°C for 2 min, 95°C for 10 min, 95°C for 15 s and 60°C for 1 min. All PCR plates were run with a no template control (NTC) and a positive control (2ng/uL *P. multocida* DNA). We tested the limits and sensitivity of the assay using serial twelve-fold dilutions, starting at a concentration of 1 ng/uL *P. multocida* DNA. The detection limit of the assay was 1x10^-8^ ng/uL. DNA concentrations were quantified by UV spectroscopy by NanoDrop spectrophotometer (NanoDrop Technologies, Wilmington, DE). A sample was classified as positive if the Ct value was 38 or less ([Corney et al. 2007](#_ENREF_6)).

**Appendix S3 Detailed methods used to measure immune traits**

Blood was sampled in heparinized syringes, and stored on ice for 1–4 h before centrifugation, and plasma was frozen at -20°C in the ﬁeld and subsequently -80°C until laboratory analysis.

Heterophils:Lymphocytes ratio (HL ratio)

Heterophils are phagocytosing cells that enter the tissues during an inflammatory response ([Parslow 1994](#_ENREF_17); [Ots et al. 1998](#_ENREF_16)). They are part of the innate immune defence, which plays an important role during the initial stages of most infections, and are the primary means of controlling bacterial infections ([Schat et al. 2014](#_ENREF_18)). Increased level of heterophils is typical during inflammation, the non-specific response to foreign invasion or tissue damage, and increased heterophil/lymphocyte ratio (H/L ratio) is known to increase in response to various stressors, including infectious diseases, starvation and psychological disturbance ([Gross and Siegel 1983](#_ENREF_8); [Maxwell 1993](#_ENREF_14); [Ots et al. 1998](#_ENREF_16)). Lymphocytes, on the other hand, are the main cell types in the adaptive immune response (i.e., B-cells and T-cells). Both cell types are highly specific and will only start proliferation once a specific antigen is recognised ([Janeway and Medzhitov 1999](#_ENREF_10)). Decreasing lymphocyte levels are indicative of immunosuppression with a concomitant increase in susceptibility to infections ([Siegel 1995](#_ENREF_20); [Ots et al. 1998](#_ENREF_16)).

Immunoglobulin Y (IgY)

We used an index of humoral immunity, quantified as levels of circulating Immunoglobulin Y (IgY) serum proteins. IgY levels are known to integrate both genotypic and phenotypic effects ([Apanius and Nisbet 2006](#_ENREF_1)), and are therefore thought to be a reliable and useful proxy of individual quality ([Guindre-Parker et al. 2013](#_ENREF_9)). IgY is the principal antibody class in birds and acts as an important first line of defence mediating a variety of protective functions through interaction with specific receptors and immune mediators ([Sugita-Konishi et al. 1996](#_ENREF_21); [Bourgeon et al. 2010](#_ENREF_5)). IgY levels are also highly responsive to changes in body condition and parasite loads ([Bourgeon et al. 2006](#_ENREF_4); [Bourgeon and Raclot 2006](#_ENREF_3); [Tomás et al. 2007](#_ENREF_22); [Bourgeon et al. 2010](#_ENREF_5)). We measured IgY using an in-house enzyme-linked immunosorbent assay that utilizes commercial anti-chicken antibody ([Martínez et al. 2003](#_ENREF_11); [Bourgeon et al. 2006](#_ENREF_4); [Bourgeon and Raclot 2006](#_ENREF_3)). This method has previously been validated for use in common eiders (as outlined in ([Bourgeon et al. 2006](#_ENREF_4))) and we read absorbance at 405nm—the change in colour measured is proportional to the IgY content of the sample and levels are expressed in arbitrary absorbance units. The mean intra- and inter-assay coefficients of variation were 1.1% and 15.1%, respectively.

Haptoglobin (Hp)

We measured the concentration of haptoglobin (mg.mL^-1^) in plasma. Haptoglobin is an acute-phase protein that scavenges haemoglobin in the event of intravascular or extravascular haemolysis. Haptoglobin is a marker of inflammation and also found in strong association with diseases that have inflammatory causes on various animal taxon including birds. To measure Hp we using a commercially available kit (phase haptoglobin, TP801; Tri-Delta Diagnostics, Morris Plains, NJ), with slight modifications to the manual method instructions provided by the manufacturer ([Matson 2006](#_ENREF_12)). More details are given in ([Berzins et al. 2011](#_ENREF_2)). Samples were run in duplicate when possible and randomized by year. Inter-plate assay variation calculated from one control sample run on each assay plate was 11.1 %.

Plasma hemolysis may be important factor when quantifying Hp levels. Recently, [Matson et al. (2012](#_ENREF_13)) showed that Hp was influenced by plasma colour (as indicated by hemolysis coded in five groups). Here we also found an effect of hemolysis on Hp (F_1,195_ = 197.8, P< 0.001). Because an index of hemolysis was not available for each sample, we thus analysed data with different subsets using a cut-off of 0.9 mg.ml^-1^ to avoid inclusion of samples with high levels of hemolysis. Results were unchanged if the cut-off was > 0.6. We also performed the analyses using a subset of individuals for which there was uncertainty on Hp values (e.g., only single measure, or a relatively high coefficient of variation); the results were unchanged.

NAb & complement

We measured the plasma levels of NAb-mediated agglutination and complement-mediated lysis by performing a hemolysis-hemagglutination assay. This assay quantifies titres of NAbs and complement-like lytic enzymes which are components of innate immunity. NAbs non-specifically bind to foreign antigens, which can neutralize infection and activate the complement system ([Ochsenbein and Zinkernagel 2000](#_ENREF_15)). Activation of the complement cascade can lead to the rupture of pathogens ([Ochsenbein and Zinkernagel 2000](#_ENREF_15)).

For the general assay procedure we first serially diluted 8 plasma samples down the long axis of a 96-well assay plate and then added a 1% rabbit red blood cell suspension into all wells (more details are given in ([Berzins et al. 2011](#_ENREF_2))). Plates were then incubated for 90 min at 37^o^C, and the long axis of the plate was tilted at a 45^o^ angle for 20 min at room temperature. At 20 and 90 min post-incubation, we scanned each plate to quantify titres of NAbs and complement activity, respectively. Samples were run randomized by year and all assay scoring was performed blindly to sample identity by LLB. The mean inter-plate assay variation was 2.7 % for agglutination titres and 14.7 % for lysis titres.

**References**

Apanius, V. and I. C. Nisbet. 2006. Serum immunoglobulin G levels are positively related to reproductive performance in a long-lived seabird, the common tern (*Sterna hirundo*). Oecologia **147**:12-23.

Berzins, L. L., H. G. Gilchrist, K. D. Matson, and G. Burness. 2011. Sex-specific effects of increased incubation demand on innate immunity in Black Guillemots. Physiological and Biochemical Zoology **84**:222-229.

Bourgeon, S. and T. Raclot. 2006. Corticosterone selectively decreases humoral immunity in female eiders during incubation. Journal of Experimental Biology **209**:4957-4965.

Bourgeon, S., F. Cruscuolo, Y. Le Maho, and T. Raclot. 2006. Phytohemagglutinin response and immunoglobulin index decrease during incubation fasting in female common eiders. Physiological and Biochemical Zoology **79**:793-800.

Bourgeon, S., M. Kauffmann, S. Geiger, T. Raclot, and J.-P. Robin. 2010. Relationships between metabolic status, corticosterone secretion and maintenance of innate and adaptive humoral immunities in fasted re-fed mallards. J Exp Biol **213**:3810-3818.

Corney, B., I. Diallo, L. Wright, G. Hewitson, A. D. Jong, P. Burrell, P. Duffy, C. Stephens, B. Rodwell, and D. Boyle. 2007. *Pasteurella multocida* detection by 5′*Taq* nuclease assay: A new tool for use in diagnosing fowl cholera. Journal of microbiological methods **69**:376-380.

Descamps, S., J. Bêty, O. P. Love, and H. G. Gilchrist. 2011. Individual optimization of reproduction in a long-lived migratory bird: a test of the condition-dependent model of laying date and clutch size. Functional Ecology **25**:671-681.

Gross, W. B. and H. S. Siegel. 1983. Evaluation of the heterophil/lymphocyte ratio as a measure of stress in chickens. Avian Diseases **27**:972-979.

Guindre-Parker, S., H. G. Gilchrist, S. Baldo, S. M. Doucet, and O. P. Love. 2013. Multiple achromatic plumage ornaments signal to multiple receivers. Behavioral Ecology **24**:672-682.

Janeway, C. A. and R. Medzhitov. 1999. Innate immunity: Lipoproteins take their Toll on the host. Current biology **9**:R879-R882.

Martínez, J., G. Tomás, S. Merino, E. Arriero, and J. Moreno. 2003. Detection of serum immunoglobulins in wild birds by direct ELISA: a methodological study to validate the technique in different species using antichicken antibodies. Functional Ecology **17**:700-706.

Matson, K. D. 2006. Are there differences in immune function between continental and insular birds? Proceedings of the Royal Society B-Biological Sciences **273**:2267-2274.

Matson, K. D., N. P. C. Horrocks, M. A. Versteegh, and B. I. Tieleman. 2012. Baseline haptoglobin concentrations are repeatable and predictive of certain aspects of a subsequent experimentally-induced inflammatory response. Comparative Biochemistry and Physiology Part A: Molecular & Integrative Physiology **162**:7-15.

Maxwell, M. 1993. Avian blood leucocyte responses to stress. World’s Poultry Science Journal **49**:34-43.

Ochsenbein, A. F. and R. M. Zinkernagel. 2000. Natural antibodies and complement link innate and acquired immunity. Immunology today **21**:624-630.

Ots, I., A. Murumägi, and P. Horak. 1998. Haematological health state indices of reproducing great tits: methodology and sources of natural variation. Functional Ecology **12**:700-707.

Parslow, T. 1994. The phagocytes: neutrophiles and macrophages. Basic and clinical immunology, 8th edn. Appleton and Lange, Norwalk, Conn:9-20.

Schat, K. A., B. Kaspers, and P. Kaiser. 2014. *Avian Immunology*. 2 ed: Elsevier.

Sénéchal, É., J. Bêty, H. Gilchrist, K. Hobson, and S. Jamieson. 2011. Do purely capital layers exist among flying birds? Evidence of exogenous contribution to arctic-nesting common eider eggs. Oecologia **165**:593-604.

Siegel, H. 1995. Stress, strains and resistance 1.

Sugita-Konishi, Y., K. Shibata, S. S. Yun, Y. Hara-Kudo, K. Yamaguchi, and S. Kumagai. 1996. Immune functions of immunoglobulin Y isolated from egg yolk of hens immunized with various infectious bacteria. Bioscience, biotechnology, and biochemistry **60**:886.

Tomás, G., S. Merino, J. Moreno, J. Morales, and J. Martínez. 2007. Impact of blood parasites on immunoglobulin level and parental effort: a medication field experiment on a wild passerine. Functional Ecology **21**:125-133.
